# Achieving Single-Cell Resolution via Desorption Electrospray Ionization Mass Spectrometry Imaging (DESI-MSI) on Different Platforms

**DOI:** 10.1021/acs.analchem.5c08032

**Published:** 2026-03-30

**Authors:** Nathan Colwell, Dan Chen, Deepti Bhusal, Zongkai Peng, Zhibo Yang

**Affiliations:** † Department of Chemistry and Biochemistry, 6187 University of Oklahoma, 101 Stephenson Parkway, Norman, Oklahoma 73019, United States; ‡ Department of Biochemistry and Physiology, University of Oklahoma Health Campus, Oklahoma City, Oklahoma 73104, United States

## Abstract

Desorption electrospray ionization (DESI) is a widely used ambient mass spectrometry imaging (MSI) technique valued for its minimal sample preparation and ability to preserve native chemical states. However, achieving single-cell resolution with DESI has been challenging due to relatively low efficiencies of molecular ionization and ion transmission at small spatial scales. Here, we present four distinct implementations that enable single-cell DESI imaging of cultured cells through a combination of optimized experimental parameters and modular hardware integration. In the first platform, a Waters system consisting of a DESI XS source and a Synapt G2-Si Q-TOF mass spectrometer was used with a customized heated ion-transfer capillary and carefully optimized key parameters, including heating temperature, sprayer-to-surface distance, and solvent flow rate, for improved desolvation and ion transmission. In the second platform, a home-built sampling and ionization setup, including a DESI XS sprayer, motorized XYZ-stage, microscope, and ion–source interface, was coupled to a Thermo LTQ Orbitrap XL mass spectrometer. In the third platform, a similar setup containing a DESI XS sprayer was integrated with a Thermo Exploris 240 Orbitrap mass spectrometer. In the fourth platform, a similar setup was coupled to a Thermo Orbitrap Fusion Lumos mass spectrometer. All four platforms allowed MSI studies of metabolites in single cells with heterogeneous populations. Integration with Orbitrap systems provided higher mass resolution and improved spatial resolution, facilitating a demonstration of DESI-based single-cell MSI. Among all four platforms, combining DESI XS source with Exploris 240 resulted in the smallest pixel size (2.7 μm × 10 μm) and largest number of detected molecular features. Together, these results establish a flexible and reproducible framework for adapting DESI across platforms for high-resolution ambient MSI and reveal distinct chemical differences between neighboring cells under native conditions.

## Graphical abstract

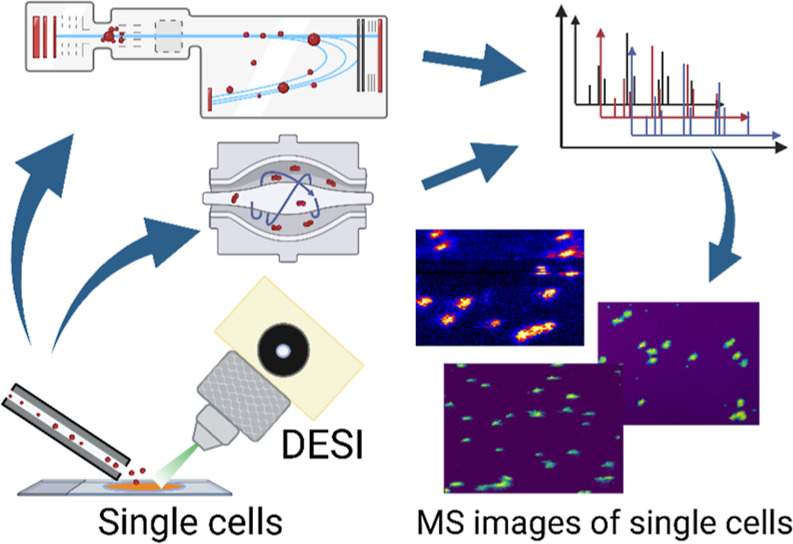

## Introduction

### Background on Mass Spectrometry Imaging (MSI)

Mass spectrometry imaging (MSI) is a transformative analytical technique that provides spatially resolved detection of metabolites, lipids, peptides, and proteins directly from biological samples, offering a detailed view of the molecular architecture within tissues. Its applications are broad and impactful across biomedical research, including elucidating metabolic reprogramming in cancer,^1^ characterizing lipid dysregulation in neurodegenerative diseases,^2^ and monitoring tissue-level responses to therapeutic interventions.^3^ MSI also enables spatially resolved biomarker discovery,^4^ allowing researchers to pinpoint molecular signatures associated with early disease progression or treatment resistance. By generating high-resolution chemical maps, MSI facilitates the study of tissue microenvironments, immune cell infiltration, and cellular heterogeneity, which are features that are often obscured in bulk analyses where spatial context is lost.^5^ This ability to correlate molecular distributions with tissue architecture and pathology provides unique insights into complex biological systems and supports the development of more precise, mechanism-based therapeutic strategies.

In practice, MSI operates by raster scanning the sample surface pixel by pixel. At each pixel, a specific ionization method is applied to ionize the molecules present. The generated ions are then transmitted through ion guides into the mass spectrometer, where they are detected based on their mass-to-charge (*m*/*z*) ratios, yielding complete mass spectra for that pixel. The collection of these spectra is then compiled to produce detailed molecular images that correlate spatial information with chemical composition. The integration of precise control of sample motion, effective ionization, and efficient ion transmission is critical for achieving the sensitivity and spatial resolution required for advanced biomedical research.^6^

MSI techniques are broadly categorized into nonambient and ambient approaches, each with distinct advantages and limitations.^4,7,8^ Nonambient techniques, such as Matrix-Assisted Laser Desorption Ionization (MALDI)^9^ and secondary ion mass spectrometry (SIMS),^10^ operate under vacuum conditions and typically require complex sample preparation. These methods generally achieve high spatial resolution and enhanced signal quality; however, the rigorous sample preparation can alter the native state of the tissue, and the vacuum environment may not capture transient molecular species effectively.^11^ In contrast, ambient ionization techniques perform analyses under atmospheric conditions with minimal sample preparation, thereby preserving the native state of the sample. Examples of ambient methods include desorption electrospray ionization (DESI),^12,13^ laser ablation electrospray ionization (LAESI),^14^ and the Single-probe.^15–20^ Although ambient approaches may sometimes exhibit inferior spatial resolution or sensitivity compared to nonambient methods, their ability to rapidly analyze samples in situ is a significant advantage in many biomedical applications.

## Overview of Previous Single-Cell MSI Studies

Single-cell imaging via MSI presents unique challenges compared to traditional tissue imaging due to the extremely small size of individual cells (e.g., ranging from 5 to 20 μm in diameter).^8,21–23^ To accurately capture the chemical heterogeneity within a single cell, the pixel size of the imaging system must be sufficiently small—often on the order of 5 to 10 μm or less—to resolve subcellular features. This stringent spatial resolution requirement places significant demands on the ionization efficiency and overall sensitivity of the mass spectrometric instrumentation. Several MSI techniques have demonstrated the capability to achieve cellular and subcellular resolutions. Among nonambient methods, techniques based on MALDI and SIMS are well recognized. MALDI has achieved pixel resolutions down to ∼1 μm through optimized laser focusing and matrix application, while SIMS offers submicron resolution, making it highly effective for detailed mapping of cellular membranes and intracellular components.^24,25^

To overcome above-stated drawbacks of vacuum-based single-cell MSI techniques, ambient approaches, such as the Single-probe^15–17,20^ and nano-DESI,^26^ have been developed to enable single-cell MSI, although they frequently require sophisticated ionization sources to attain the necessary spatial resolution. These examples underscore the trade-offs between spatial resolution, molecular coverage, and system complexity in developing single-cell MSI methodologies as well as highlight the need for a commercially available, ambient imaging method for single cells.

### The Potential and Challenges of DESI for Single-Cell Imaging

DESI is widely recognized for its effective applications of direct MS analysis of sample surfaces under ambient conditions, offering the advantages of minimal sample preparation and preservation of the sample’s native state. The wide application of DESI-MSI stems from these operational benefits and the relative ease with which it can be implemented. However, using DESI-MSI for single cell studies are generally rare, largely due to its limited spatial resolution. Recent advancement in DESI techniques has greatly improved its spatial resolution. Using carefully optimized standard system, Waters DESI-XS ion source coupled with a Cyclic ion mobility mass spectrometer, Zhang et al. showed DESI’s capabilities at the single-cell level by utilizing ultralow solvent flow rates of 150 nL/min.^27^ To overcome the challenges of solvent spray instability at such low flow rate, they coupled the sprayer to a C18 column to increase the back pressure.

With a new area of DESI imaging unlocked, further improvements are needed to enable its capabilities of single cell studies on different systems. A key challenge in this context is the low ion signals typically generated from small amounts of analytes present in individual cells. Ionization efficiency in DESI-MSI can be affected by multiple factors, such as the sample’s surface properties, the spray angle and distance, the voltage applied to the spray, the solvent composition, and the solvent flow rate, which can lead to variability in signal intensity and complicate quantitative analyses.^27–29^ Previous work by Venter et al. has outlined strategies to enhance ionization efficiency in DESI through optimization of spray parameters, solvent dynamics, and surface interactions, highlighting how these factors collectively improve desorption and ion yield under ambient conditions.^30^

Additionally, analyte ions must be efficiently transferred from the sample surface into the mass spectrometer through an ion transfer capillary and other ion optics. Signal loss can arise from ion neutralization, adsorption of ions to internal surfaces, or incomplete transfer of ionized species, all of which diminish overall signal intensity.^31,32^ Increasing ionization efficiency is regarded as an effective strategy for improving sensitivity. A recent study by Zickuhr et al.^33^ demonstrated that increasing the capillary temperature from 150 to 450 °C led to as much as a 1.8-fold increase in signal intensity. Building on these findings, we aimed to improve the detection sensitivity of DESI experiments by optimizing multiple experimental conditions, including capillary heating, spray geometry, and solvent parameters, in order to achieve single-cell MSI.

Further developing DESI in cellular ranges would allow researchers to exploit its ambient nature to perform in situ analysis at the level of individual cells—unlocking detailed insights into cellular metabolism and heterogeneity that are currently out of reach with existing methods. Another significant advantage of DESI is its popularity, which greatly facilitates its adoption by the MS community. If DESI can be adapted to readily achieve single-cell resolution, it promises to bridge the gap between high-resolution molecular imaging and user-friendly, widely accessible instrumentation, thereby broadening the scope of applications in biomedical research.

### Novel Contributions and Research Objectives

This work demonstrates DESI-MSI of cultured individual cells using commercially available instruments with modifications. Unlike nanoDESI and Single-probe methods, which require microscale extraction of cellular contents by the liquid bridge at the probe tip, DESI-based methods are less sensitive to sample surface topology and have no clogging issues of fluidic devices, providing more robust measurement with higher tolerance of sample surface. The noncontact extraction of DESI also has less influence on sample surface, allowing for subsequent analysis sensitive to sample integrity such as spatial transcriptomics.^34^

In this study, we demonstrated four different systems for single-cell DESI imaging of cultured cells. The first implementation employs a heated ion transfer capillary on a Waters DESI XS/Synapt G2-Si QTOF system with minor modification. Our results were achieved through hardware modification and instrument optimization, demonstrating the potential of using the existing, older generations of mass spectrometers for single cell studies.^27^ The second implementation couples the Waters DESI-XS sprayer to a Thermo Scientific LTQ Orbitrap XL mass spectrometer, which has been successfully used for the Single-probe MSI studies in our lab.^16,20^ The third implementation integrates the same DESI XS sprayer with a Thermo Scientific Exploris 240 Orbitrap, a newer-generation platform with further improved performance. The fourth implementation integrates the DESI XS sprayer with a Thermo Scientific Orbitrap Fusion Lumos Tribrid mass spectrometer. This work establishes DESI MSI as a practical, high-performance tool for ambient single-cell molecular analysis and sets the stage for its broad adoption in biomedical research. Particularly, the combination of DESI source with Orbitrap mass spectrometers takes advantage of the Orbitrap’s high mass accuracy and resolving power to distinguish closely related metabolites at the single-cell level. In addition, we developed a modular, machinable sprayer mount compatible with both legacy and later generations of Orbitrap instruments, including the Tribrid, Exploris, and Astral series, which positions this setup for broad accessibility and future scalability.

## Materials and Methods

### Sample Preparation

OVCAR-8 human ovarian cancer cells were cultured in RPMI complete media (RPMI-1640 supplemented with 10% fetal bovine serum and 1% penicillin–streptomycin) under standard conditions until reaching approximately 80% confluency. Prior to cell transfer, 6-well plates (cat.no:3471, CORNING, Kennebunk, ME, USA) were prepared by placing a gridded glass coverslip (cat.no:10817, ibidi, Gräfelfing, Germany) into each well and adding 4 mL of complete media. Approximately 1 × 10^5^ cells were then seeded into each well. The plates were incubated for 12 h to allow for adequate cell attachment and spreading on the coverslips (Figure S1). Following incubation, the coverslips were carefully removed and washed with an ammonium formate solution (143 mM) to remove excess salts, thereby reducing potential interferences during mass spectrometry analysis. The washed coverslips were subsequently mounted on glass slides for DESI-MS imaging.

## Instrumentation and Experimental Setup

### Optimized Waters DESI XS/Synapt G2-Si System

First, we prepared the surface coated with dried cell lysates. To do this, we cultured OVCAR-8 cells, prepared cell lysate, and coated multiple spots on a PTFE-coated glass slide with 10 μL of cell lysate on each spot, then allowed the samples to air-dry (Figure S2). OVCAR-8 human ovarian cancer cells were chosen as a representative epithelial cancer model.

Second, we performed systematic experiments to optimize the temperature of the ion transfer capillary. Specifically, we wrapped the heating wire (22 gauge, Kanthal, Bethel, CT, USA), covered by a fiberglass mesh sleeve, around the ion transfer capillary (Figure 1). This assembly was connected to a thermocouple to monitor the temperature and a universal power supply (model: LGY-363000, Dong Guan Shi He Yu Tech, Dongguan, China), which allowed us to apply varying voltages to reach and maintain specific temperatures. We adjusted the voltage applied from the power supply to achieve a series of target temperatures: room temperature, 250, 275, 300, 325, 350, 375, 400, and 425 °C. Third, for each temperature, the DESI XS stage was manually repositioned to manipulate the distance between the sprayer and the spot coated with dried cell lysate on the slide. In general, a short distance can improve ion intensities, whereas an excessively short distance may cause the sprayer to scratch the sample. Last, we optimized the flow rate of solvent (methanol 95%/water 5% + formic acid 0.1%) delivered by the LC pump system (no column) with a voltage of 0.85 kV. To determine the optimal values for each parameter, we monitored the total ion current (TIC) and selected the condition that produced the highest normalized level (NL) signal. Based on this approach, the optimized experimental parameters were determined to be an ion transfer capillary temperature of 375 °C, a distance of 1.2 mm between sprayer and surface, and a flow rate of 0.5 mL/min. Other parameters included a scan rate of 20 μm/s, mass range of 100–1200 *m*/*z*, TOF mode, 20 k mass resolution, and positive ion mode.

**Figure 1:**
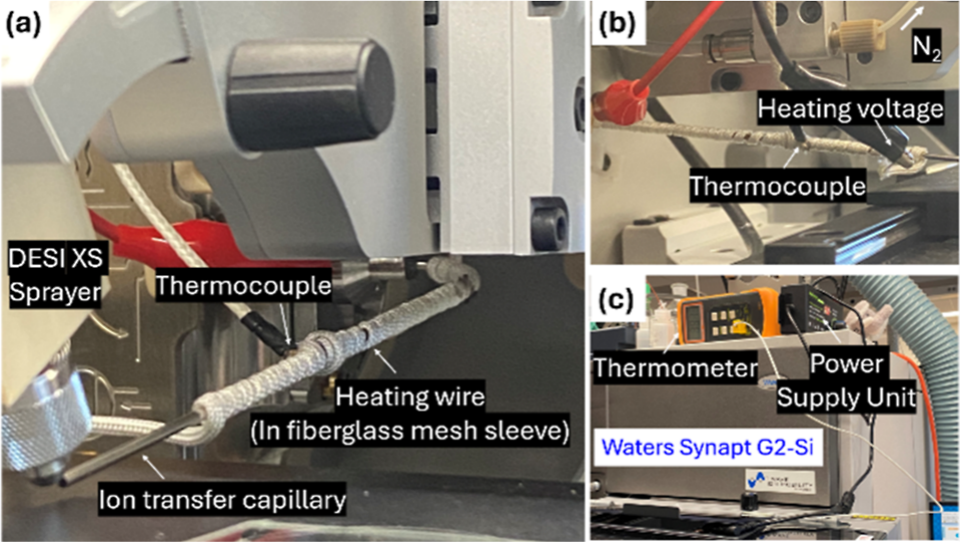
Heating ion capillary setup on Synapt G2-Si. Experimental configuration illustrating the integration of a heating element with the ion transfer capillary. (a) Heating wire encased in a fiberglass mesh sleeve and wrapped around the ion capillary. (b) Photograph showing the assembled setup, including the thermocouple and alligator clips connecting the heating wire to the power supply unit (PSU). (c) Photograph displaying the digital thermometer and PSU used to monitor and control capillary temperature.

### Coupling DESI XS Sprayer with LTQ Orbitrap XL

To enable coupling with the Thermo LTQ Orbitrap XL mass spectrometer, a Waters DESI XS sprayer was mounted onto an integrated system, which consists of an ion source interface flange, motorized XYZ-stage, digital microscope, and an optical breadboard, providing precise sample control, sprayer positioning, and visual monitoring (Figure 2). A customized ion transfer tube with extended length and curved shape was used to substitute the standard ion transfer tube (Figure S3). This integrated system was adopted from the existing system for single-cell MS and MSI studies using the Single-probe setup^15–17,20,35–38^ with minor modifications. During DESI-MSI experiment, nitrogen was supplied as a sheath gas to support stable spray formation, while solvent (acetonitrile 95%/water 5% with formic acid 0.1%) was delivered from a Waters nanoAcquity UPLC system (150 nL/min). To maintain stable spray conditions at low solvent flow rates, a C18 column (Waters, part no. 186009259, serial no. 04733426016309) was incorporated into the LC system. This addition provided additional back pressure to stabilize the spray, demonstrated by Zhang et al.^27^ Both the ionization voltage (0.85 kV) and nitrogen nebulization gas (∼12 psi Using the sheath gas from the LTQ Orbitrap XL) were sourced directly from the mass spectrometer housing outlet and applied on the DESI-XS sprayer. Additionally, the automated XYZ-stage system, which was controlled using a customized LabVIEW software, allowed to raster the sample with high spatial precision, contributing significantly to the ability to achieve single-cell resolution.^39^ Other mass spectrometer settings include the mass resolution of 60 K (at *m*/*z* 200), one microscan, a maximum injection time of 100 ms, and the use of an automatic gain control (AGC).

**Figure 2:**
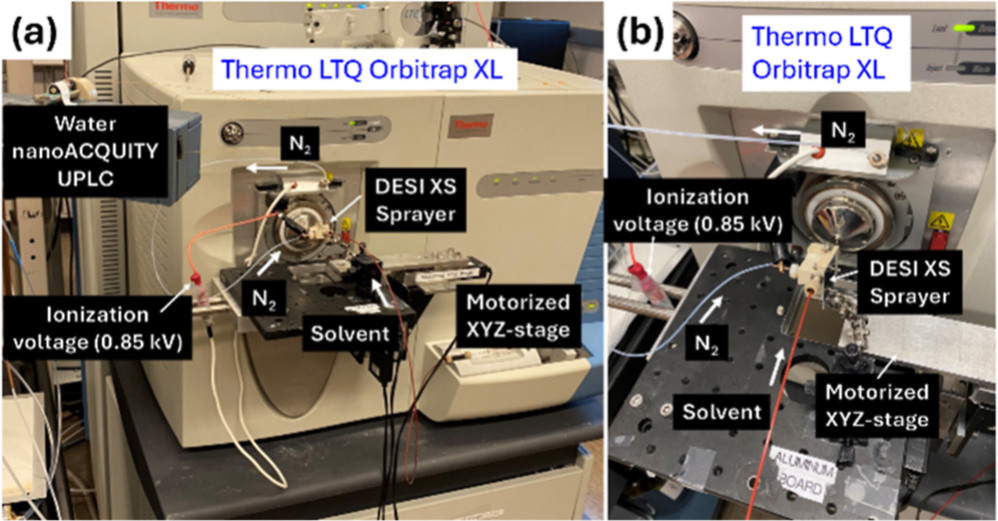
Waters DESI XS sprayer coupled to Thermo LTQ Orbitrap XL mass spectrometer. (a) Photo showing DESI XS sprayer mounted to an XYZ-stage affixed to a breadboard, with the connections for N_2_ sheath gas and voltage supply from the mass spectrometer as well as solvent delivery from a Waters nanoAcquity UPLC system. A custom-modified inlet housing enables coupling of the DESI XS sprayer to the LTQ Orbitrap XL. (b) Zoomed-in photo of the complete setup, highlighting the DESI XS sprayer and automated XYZ-stage.

### Coupling DESI XS Sprayer with Exploris 240

To enable DESI MSI studies of single cells using newer generations of Orbitrap mass spectrometers, we transitioned to the Thermo Scientific Exploris 240, aiming to leverage its advanced performance for high-resolution DESI-MS imaging. To integrate the DESI XS sprayer with this instrument, we machined and converted a Thermo FAIMS (field asymmetric ion mobility spectrometry) interface frame to serve as a custom mounting platform (Figures S4 and S5). The housing was modified to accommodate an optical breadboard, onto which the motorized XYZ-stage and digital microscope were installed, enabling precise sample motion control, sprayer adjustment, and compatibility with the instrument’s standard source geometry. A customized ion transfer tube was used to substitute the standard part to enable efficient ion transfer (Figures S6 and S7). Nitrogen gas was supplied from the building’s centralized system, regulated to 12 psi, and connected to the sprayer’s sheath line to maintain spray stability. The spray solvent (acetonitrile 95%/water 5% + formic acid 0.1%) was delivered via a nanoLC system (750 nL/min) equipped with a C18 column (Waters, part no. 186009259, serial no. 04733426016309) and an ionization voltage of 0.85 kV. Raster scanning was performed using an automated XYZ-stage to ensure controlled motion and high spatial sampling density (Figures 3 and S4, S5).

**Figure 3:**
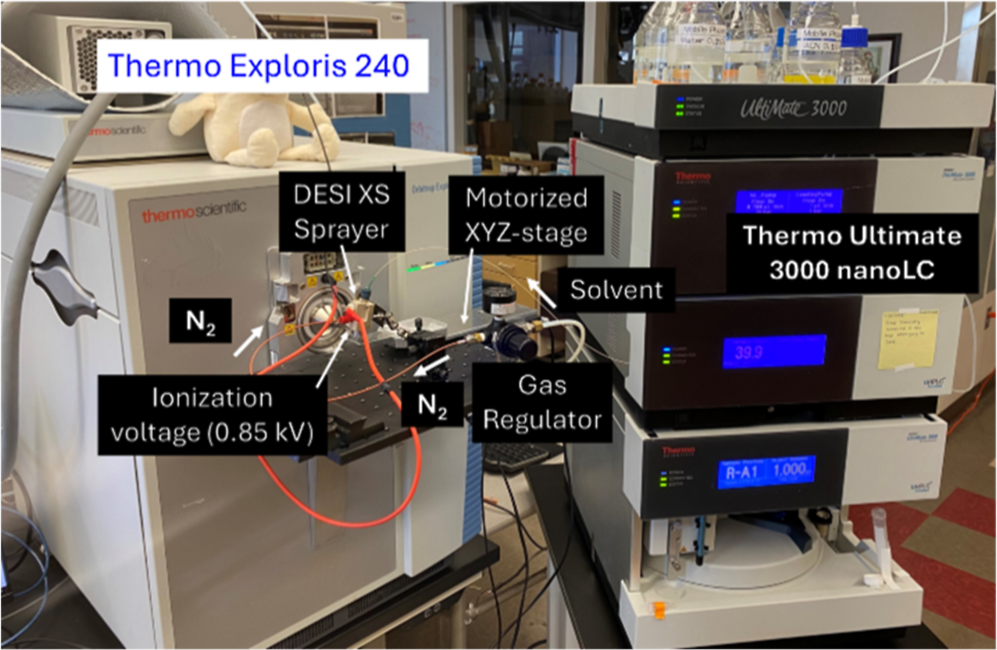
Waters DESI XS Sprayer coupled to Thermo Exploris 240. The DESI XS sprayer is integrated with the Thermo Exploris 240 using a modified FAIMS interface, allowing mounting via an XYZ-stage secured to a breadboard. Solvent delivery is provided by a Thermo Ultimate 3000 nanoLC system, while N_2_ gas, regulated to ∼12 psi, is supplied from the building line to support the sprayer.

Since FAIMS interfaces are common across several Thermo Scientific platforms, including the Orbitrap Exploris, Orbitrap Tribrid, and Astral series, this configuration provides a broadly adaptable and scalable blueprint for retrofitting DESI sources onto compatible instruments. Due to complex instrumentation, we were unable to couple our current setup, which consists of the DESI XS source and XYZ-stage system, with the standard FAIMS interface. However, we note that DESI-FAIMS integrations have been previously utilized, illustrating the potential of gas-phase ion separation to reduce chemical noise and improve molecular selectivity in ambient imaging.^40^ We expect that additional instrumentation in the future can potentially improve the performance of our DESI/Orbitrap systems in single cell MS studies.

### Coupling DESI XS Sprayer with Orbitrap Fusion Lumos

To further extend compatibility of the DESI XS platform to Orbitrap Tribrid instruments, the Waters DESI XS sprayer was interfaced with a Thermo Scientific Orbitrap Fusion Lumos mass spectrometer. The complete setup, including the modified FAIMS interface, optical board, DESI XS source, XYZ-stage system, gas regulator, and digital microscope, was adopted from the DESI/Exploris 240 system and coupled to the Orbitrap Fusion Lumos mass spectrometer with minor modifications (Figure 4). A customized curved ion transfer tube (Figure S7) was used to replace the standard inlet to improve geometric alignment between the DESI plume and the heated ion transfer capillary. Nitrogen gas was supplied from the instrument housing to the sheath gas line (∼12 psi) to maintain stable spray formation, and the electrospray potential (0.85 kV) was applied directly from the mass spectrometer outlet to the DESI sprayer. The same solvent, nanoLC system, and C18 column were adopted from the DESI/Exploris system. Experiments were operated at a low solvent flow rate (700 nL/min).

**Figure 4:**
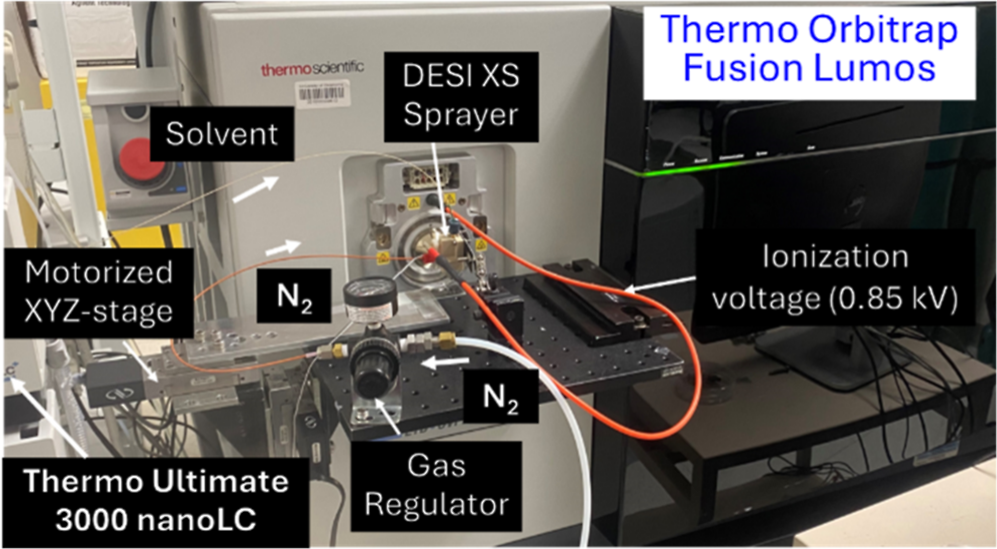
Waters DESI XS Sprayer coupled to Thermo Orbitrap Fusion Lumos. The DESI XS Sprayer is integrated with the mass spectrometer using a modified FAIMS interface, allowing mounting via an XYZ-stage secured to a breadboard. Solvent delivery is provided by a Thermo Ultimate 3000 nanoLC system, while N_2_ gas (∼12 psi) is supplied for the sprayer.

### Data Processing

MSI data acquired from the modified Waters platform were processed using Waters Microapps to generate ion images with tentative annotations. Data obtained from the Orbitrap-based configurations were processed with METASPACE, which provided tentative annotations for the observed molecular profiles.^41^ These processing tools enabled the visualization of spatially resolved chemical information and facilitated comparative evaluation of the imaging performance across the different experimental setups.

MS/MS analysis of ions with relatively high abundances were conducted at the single-cell level using the following parameters on the DESI XS/LTQ Orbitrap XL: HCD mode, 1 μL/min flow rate, mass resolution of 60 k (at *m*/*z* 200), ionization voltage of 0.85 kV in positive ion mode, one microscan, a maximum injection time of 100 ms, and collision energy ranging from 15 to 25 NCE (Normalized Collision Energy). The common ions were also detected using the Exploris 240 system and used to construct MS images. However, the intensities of target ions observed on the Waters setup were inadequate for MS/MS analysis.

## Results

The DESI-MSI results are organized corresponding to the four configurations: the modified Waters platform and the DESI XS sprayer coupled with three Thermo Orbitrap systems: LTQ Orbitrap XL, Exploris 240, and Fusion Lumos . Both methanol and acetonitrile-based solvent systems were evaluated in this work, as each is commonly employed in DESI-MSI. Comparable signal quality and MS image quality were obtained across configurations. Brightfield microscope images containing cells and grids were spatially correlated with MS images to locate single cells in DESI-MS images (Figure S2). Although numerous ions were detected in each experiment (Supporting Information), only representative *m*/*z* features are displayed in the figures to concisely illustrate the spatial distribution of key molecular signals at the single-cell level.

### Modified Waters DESI XS/Synapt G2-Si System

Upon acquiring the optimized experimental conditions, we conducted DESI-MSI studies to achieve single-cell resolution. A pixel size of 10 μm × 10 μm was used to achieve high-quality MS images with spatially resolved single cells. The pixel size for the Waters DESI XS/Synapt G2-Si system (10 μm × 10 μm) was set through the instrument’s High Definition Imaging software (HDI).^42^ MSI data of individual pixel (Figure S8) were used to construct MS images using HDI. Figure 5a shows a photo of attached cells that were taken using a brightfield microscope (cat.no: 12575252, Fisher Scientific, Waltham, MA, USA) at 20× magnification. MS images obtained from this system with 10 μm × 10 μm pixel size that show the abundances of select ions, tentatively annotated with Waters MSI Analyte Browser. MS images of selected ions were illustrated using heatmap: darker areas indicate lower abundance, and brighter areas indicate higher abundance (Figure 5b–f). Excellent correlations of single cells’ locations can be observed by comparing the micrograph and MS images. Numerous molecular signals were colocalized within the same individual cells, providing confident detection of single cells. The ions displayed in the figures are representative features selected for visualization. The detected ions at *m*/*z* 428.22, 400.03, 430.22, and 446.29 likely correspond to lipid-related fragments, and their assignments are not provided due to complex cellular contents, complicated fragmentation mechanisms, and low mass resolution (20 k). It is worth noting that cells with heterogeneous molecular abundances were observed. For example, the spatial distributions of *m*/*z* 428.22 (Figure 5b) and *m*/*z* 430.22 (Figure 5e) are similar, whereas *m*/*z* 400.03 (Figure 5c), *m*/*z* 430.22 (Figure 5d), and *m*/*z* 444.27 (Figure 5f) share more similarities.

**Figure 5:**
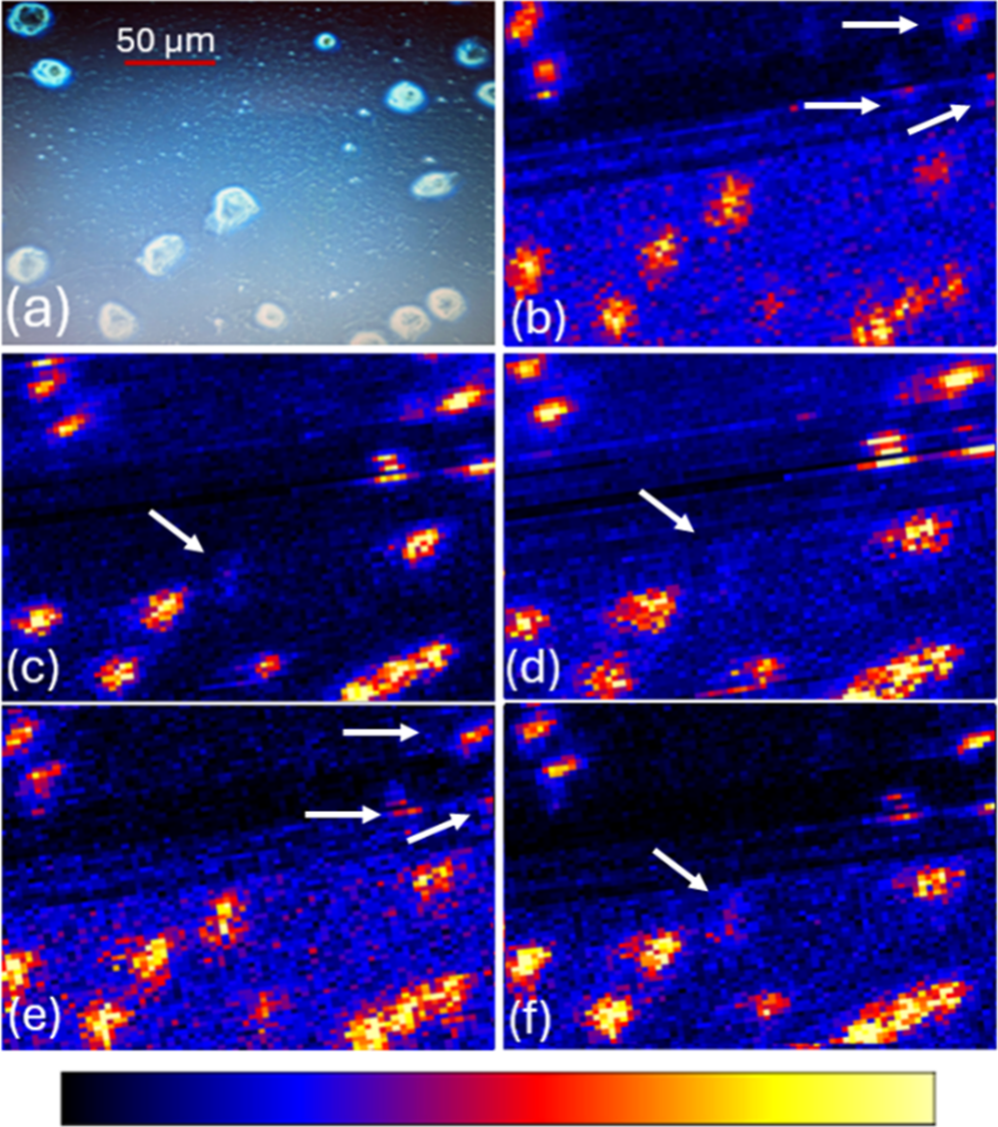
Comparison of a micrograph and MS images obtained using a Waters DESI XS/Synapt G2-Si system. (a) OVCAR-8 cells observed under brightfield microscope (20× magnification). DESI-MS images (10 μm × 10 μm pixel size) of selected ions: (b) *m*/*z* 428.22, (c) *m*/*z* 400.03, (d) *m*/*z* 430.22, (e) *m*/*z* 446.29, and (f) *m*/*z* 444.27. Arrows indicate cells with relatively low abundances of target ions.

Although a finer pixel size of 5 μm × 5 μm has been tested, we were unable to obtain improved quality of MS images, likely due to inadequate signal intensities from smaller sampling areas using our current mass spectrometer.

### Waters DESI XS/Thermo LTQ Orbitrap XL Mass Spectrometer System

Using mass spectrometers with high mass accuracy and resolving power, e.g., Orbitraps, can provide essential information for differentiating closely related molecular species in single cells.^43^ With minor modifications, we adopted our existing Single-probe setup, which is routinely used for single-cell MS and MSI studies, for the Waters DESI XS sprayer, leveraging the Orbitrap’s capabilities to enhance molecular specificity and reduce isobaric interference.^43^ This platform was used successfully to acquire MS images with markedly sharper contrast and improved signal-to-noise (Figure 2) compared to the modified Waters DESI XS/Synapt G2-Si system.

The pixel size of MS images was estimated as 6.3 μm × 10 μm (Supporting Information). In raster imaging experiments, such as using DESI techniques, the pixel dimension along the scan direction is governed by the sample stage velocity and MS data acquisition rate, whereas the orthogonal dimension is determined by the step size between adjacent raster lines. Pixel size needs to be determined based on the trade-off of multiple factors, including the target spatial-resolution, sample size, overall experimental time as well as the detection sensitivity, data acquisition speed, and resolution of mass spectrometers.

MS/MS analysis of multiple representative ions from single cells were conducted for structure identification (Figures S10–S16). Similar to the results obtained from the DESI XS/Synapt G2Si system, cells with heterogeneous molecular abundances were observed using this platform (Figure 6).

**Figure 6:**
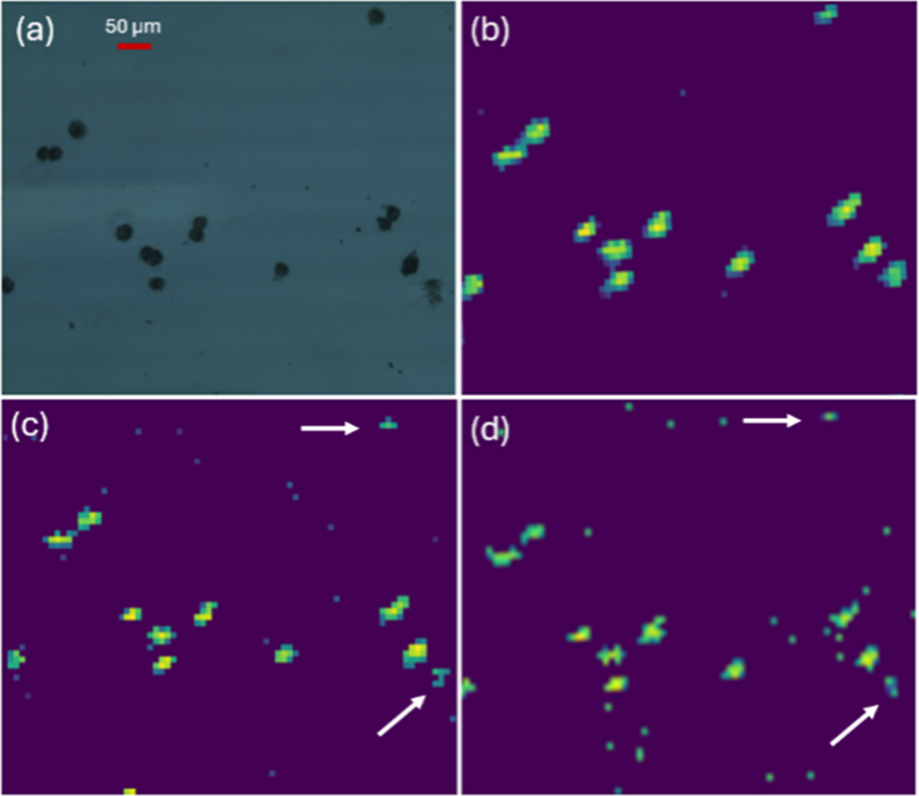
Comparison of a micrograph and MS images obtained using Waters DESI XS/Thermo LTQ Orbitrap XL. (a) Brightfield microscopy image of OVCAR-8 cells captured at 20× magnification. DESI-MS ion images (6.3 μm × 10 μm pixel size) of selected ions: (b) [PC 36:2 + H]^+^ (*m*/*z* 786.598), (c) [PC 34:1 + H]^+^ (*m*/*z* 760.583), and (d) [PC 36:2 + Na]^+^ (*m*/*z* 808.581). Each panel corresponds to a specific ion tentatively annotated using METASPACE. Arrows indicate cells with relatively low abundances of target ions.

### Waters DESI XS Sprayer Coupled with Thermo Orbitrap Exploris 240 Mass Spectrometer

Newer generations of Orbitrap technologies offer increased sensitivity, higher mass resolution, and faster data acquisition speed, which are factors that further refine spatial resolution and reproducibility in single-cell MSI. Using the integrated platform consisting of a Waters DESI XS sprayer and Thermo Scientific Exploris 240 mass spectrometer, we obtained even more detailed chemical maps, which possess improved definition of molecular features and enhanced detection of analytes with a remarkable pixel size of 2.7 μm × 10 μm (Figure 7). Each selected ion seems to possess different distribution features in single cells. Importantly, this plug-and-play interface is compatible with the latest generation of Thermo mass spectrometers, extending the reach of this DESI single-cell imaging setup across the Orbitrap product line.

**Figure 7:**
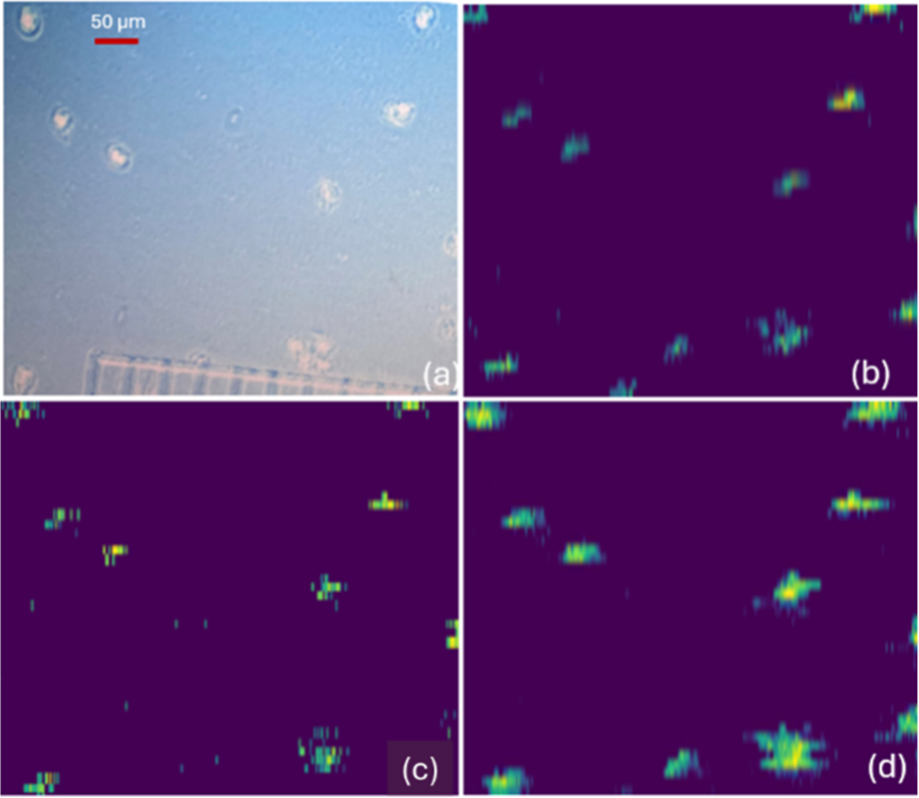
Comparison of micrograph and MS images obtained using DESI XS/Thermo Exploris 240. (a) Brightfield microscopy image of OVCAR-8 cells captured at 20× magnification. DESI-MS ion images (2.7 μm × 10 μm pixel size) of selected ions: (b) [PC 34:1 + H]^+^ (*m*/*z* 760.572), (c) [PC 18:0 + H]^+^ (*m*/*z* 524.371), and (d) [PC 36:2 + H]^+^ (*m*/*z* 786.601). Each panel corresponds to a specific ion tentatively annotated using METASPACE.

### Waters DESI XS/Thermo Orbitrap Fusion Lumos Mass Spectrometer System

To further investigate single-cell imaging performance using a Tribrid platform, the Waters DESI XS sprayer was coupled with a Thermo Scientific Orbitrap Fusion Lumos mass spectrometer. MS images of OVCAR-8 cells were obtained with the pixel of 5.5 μm × 10 μm (Figure 8). Multiple representative ions were detected within individual cells, demonstrating reliable sampling of cellular material while maintaining spatial fidelity. Compared with the DESI XS/Exploris 240 configuration, this platform produced MS images with a slightly larger pixel size, likely due to the outperformance of Exploris 240 mass spectrometer optimized for metabolomics studies. However, the availability of other fragmentation techniques, including ETD and UVPD, on a tribrid mass spectrometer enables versatile, high-confidence structural identification such as determination of double bonds in unsaturated lipids and fatty acids.

**Figure 8:**
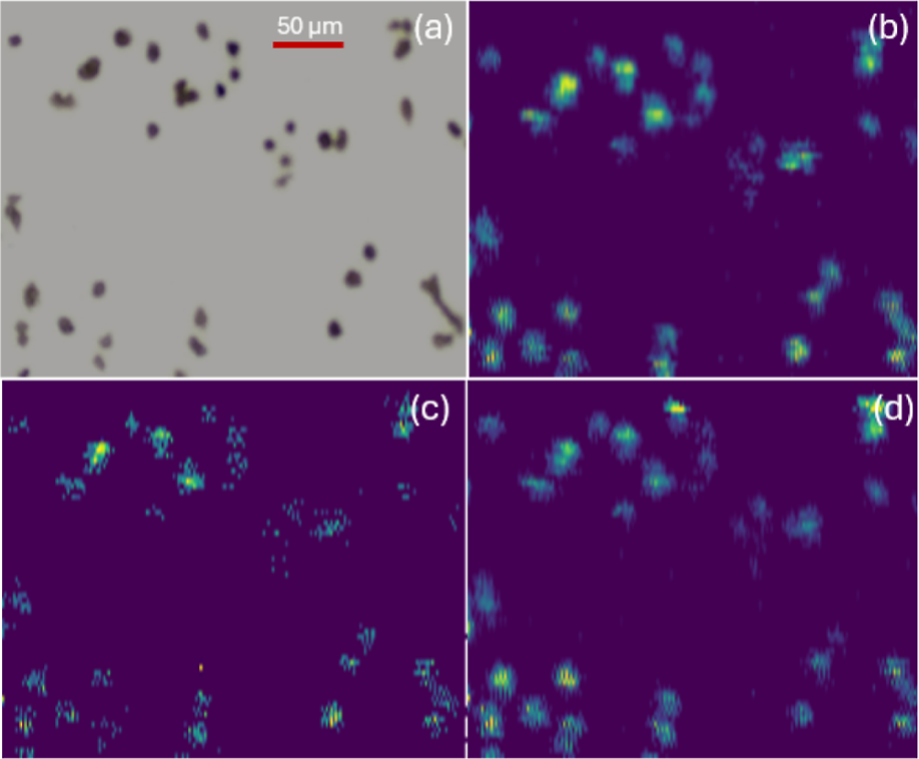
Comparison of micrograph and MS images obtained using DESI XS/Thermo Lumos. (a) Brightfield microscopy image of OVCAR-8 cells captured at 20× magnification. DESI-MS ion images (5.5 μm × 10 μm pixel size) of selected ions: (b) [PC 34:1 + H]^+^ (*m*/*z* 760.572), (c) [PC 18:0 + H]^+^ (*m*/*z* 786.583), and (d) [PC 36:2 + H]^+^ (*m*/*z* 808.560). Each panel corresponds to a specific ion tentatively annotated using METASPACE.

### Limit of Detection (LOD) of DESI XS/Thermo Orbitrap Lumos System

To evaluate analytical sensitivity of the DESI XS/Orbitrap configurations, the limit of detection (LOD) was measured using the DESI XS/Orbitrap Lumos as the representative system (Supporting Information). A series of solutions containing caffeine or reserpine at different concentrations were deposited onto PTFE-coated slides, dried, and then measured by this system. A 2.5 μL droplet of sample solution was deposited on each spot, and the resulting surface loadings were calculated based on the sample amount and surface area. The LOD was defined as the lowest concentration producing a signal equal to the average intensity of the blank plus three times the standard deviation of the blank.^44,45^ Our results indicate that the LODs were 4.9 and 7.5 ng/mL for caffeine and reserpine, respectively (Figure S9). Our results are slightly higher than those reported values in a recent study using DESI coupled to a Thermo Q-Exactive Plus mass spectrometer: 2.3 and 0.6 ng/mL for caffeine and reserpine, respectively.^46^

### Pixel Size Comparison Across MSI Techniques

The spatial resolution of MSI techniques based on MALDI and SIMS is generally defined by the pixel size of laser and ion beam, respectively. MALDI MSI methods, currently the most widely adopted technique for single-cell imaging, routinely employs pixel sizes of 5–10 μm in standard workflows, with specialized MALDI platforms capable of reaching 1–2 μm under optimized vacuum and matrix deposition conditions.^47–50^ SIMS MSI achieves much smaller pixel sizes (0.2–1 μm) under vacuum conditions, but its high-energy ion beam causes extensive fragmentation of larger biomolecules, limiting analyses primarily to small molecules and elemental species.^51,52^

Different from these vacuum-based techniques, ambient MSI methods commonly uses pixel size, which is defined by the distance the sample travels within the data acquisition time of each mass spectrum, to reflect the spatial resolution. For example, nano-DESI and Single-probe MSI report pixel sizes of approximately 8–10 μm (using a shear force probe) and 8.5 μm, respectively.^15–17,53–55^ The pixel dimensions achieved with our DESI-MSI configurations are therefore comparable to those routinely used in MALDI single-cell experiments and align with other ambient approaches, demonstrating DESI’s ability to deliver high sampling density while maintaining an accessible, minimal-preparation workflow.

## Discussion

### Interpretation of Results

Historically, DESI has been applied effectively to tissue-level imaging, but its extension to single-cell resolution has been generally hindered by issues such as signal loss during ion transmission. Our work addresses these challenges by implementing modifications that enhance ion transmission and optimize the overall ionization process.

The dominance of phosphatidylcholine signals in positive-mode DESI is expected because these lipids are major structural constituents of eukaryotic cell membranes. At single-cell spatial scales, simultaneous detection of multiple cellular lipids serves as confirmation that cellular compounds are being sampled rather than surface residue or background contamination. In addition, variations in the relative abundances of certain lipids among adjacent cells were observed, likely indicating heterogeneity of molecular profiles among different cells.

The MS imaging data clearly demonstrates that our modifications and optimizations to the DESI systems significantly improve single-cell resolution. In our first configuration, the introduction of a heated ion transfer capillary on the Waters Synapt G2-Si mass spectrometer resulted in enhanced desolvation, which in turn improved ion transmission. This modification increased ion transmission, which is critical when analyzing the minute chemical differences present within individual cells. The improved signal quality confirms that heating can effectively mitigate the signal loss observed in this system and enable its capability for single cell MSI studies.

Our second configuration, which involved coupling an optimized Waters DESI XS sprayer with a Thermo LTQ Orbitrap XL, leveraged the superior mass accuracy and resolving power of the Orbitrap platform. These attributes are intrinsic to Orbitrap-based analyzers than can generally surpass QTOF systems in resolving closely spaced or isobaric ions, thereby improving the distinction of chemical features at the single-cell level.^56^ This setup allowed us to provide a more detailed and nuanced molecular profile of each cell. The high resolving power of the LTQ Orbitrap was particularly beneficial in distinguishing subtle differences in ion signals that are essential for understanding cellular heterogeneity.

The third configuration, integrating the DESI XS sprayer with a Thermo Scientific Exploris 240 Orbitrap, built upon the improvements observed with the LTQ Orbitrap. The newer generation Exploris 240 offered further enhancements in sensitivity, mass resolution, and data acquisition speed.^57^ These improved features were translated into even more refined spatial resolution and reproducibility in our MSI studies. Compared with LTQ Orbitrap XL instrument, Exploris 240 can further improve the quality of MSI results with higher spatial resolution as demonstrated using the same representative ion (Figure S15).

In the fourth configuration, the DESI XS sprayer was coupled to a Thermo Scientific Orbitrap Fusion Lumos Tribrid mass spectrometer. The ability to maintain single-cell spatial fidelity and stable data acquisition on this system indicates that the compatibility of DESI with a tribrid Orbitrap. Importantly, versatile fragmentation techniques, such as ETD and UVPD, on a tribrid mass spectrometer provide unique capabilities for more confident structural identification, such as locating double bonds in unsaturated lipids and fatty acids, at the single-cell level.

### Platform Compatibility and Scalability

One of the most significant engineering advantages of our approach is its modularity. By combining the modified ion source interfaces with commercially available components (i.e., motorized stage system, digital microscope, and optical board), the standard DESI XS sprayer can be coupled to many models of mass spectrometers. In the current studies, the DESI XS sprayer was tested with both legacy (LTQ Orbitrap XL) and later generation (Exploris 240 and Fusion Lumos) Orbitraps. In particular, a standard FAIMS frame was modified to accommodate the DESI sprayer and other components. This integrated setup is compatible with virtually any Thermo Scientific instrument that accepts a FAIMS module, such as Tribrid, Exploris, and Astral series, for DESI MSI studies with high spatial resolution, mass resolution, and sensitivity.

### Limitations and Implications for Future Directions

Despite the promising advancements reported here, several limitations remain. Overall, instrument modification and machining need to be carried out by experienced researchers, barricading access by general users. Although heating the ion capillary of Waters Synapt G2-Si system significantly improved single-cell imaging performance, undesired ion fragmentation may occur at elevated temperatures, introducing potential artifacts. Coupling the commercial DESI XS source with Orbitrap instruments provided numerous benefits for MSI studies. It is worth noting that Waters DESI XS source is only available for users who already have Waters DESI XS-MSI systems, posing barriers to general adoption of our design. Constructing the integrated DESI XS sprayer/Orbitrap systems in the current work is only for research purposes. However, alternative DESI instrumentation, such as DEFFI, can be further improved and potentially implemented for MSI studies of single cells.^58^ It has been demonstrated that coupling DESI with FAIMS can improve MSI quality of proteins on tissue slices and reduce the noise and background.^12^ Potentially, a Thermo FAIMS setup can be added on the top of this home-built DESI XS/Orbitrap interface with additional instrumentation, allowing us to take advantage of FAIMS for further increased MS detection sensitivity. However, this implementation requires more complex instrumentation. Nevertheless, the traveling-wave ion mobility spectrometry (TWIMS) function of certain models of Waters systems enable DESI to be combined with ion mobility separation in single cell studies. While our current study demonstrates reproducible single-cell resolution with OVCAR-8 cells, validation across a broader range of cell types and biological tissues will be essential to fully establish the generalizability of the method.

The successful adaptation of DESI for single-cell imaging has far-reaching implications for various fields, including cellular biology, pharmacology, and pathology. By enabling high-resolution molecular mapping at the single-cell level, researchers can gain unprecedented insights into cellular heterogeneity and metabolic gradients within tissues. This level of detail is crucial for the identification of novel biomarkers and the development of targeted therapies, especially in complex diseases such as cancer. Furthermore, the ambient nature of DESI supports rapid, in situ analysis, potentially facilitating real-time diagnostics and personalized medicine applications. The adaptability of our approach suggests that similar modifications could be applied to other ambient ionization techniques, broadening the scope and impact of MSI in biomedical research.

## Conclusion

This study demonstrates the successful extension of DESI to single-cell imaging through four distinct implementations: (1) a standard Waters DESI XS/Synapt G2-Si QTOF system with a customized heated ion transfer capillary and optimized experimental conditions, (2) a Waters DESI XS/Thermo LTQ Orbitrap XL, (3) a Waters DESI XS/Thermo Scientific Exploris 240 Orbitrap, and (4) a Waters DESI XS/Thermo Scientific Orbitrap Fusion Lumos. The heated ion transfer capillary enhanced ion transmission, whereas Orbitrap mass spectrometers provided improved mass resolution and sensitivity. These modifications effectively address the limitations associated with traditional DESI setups, particularly the signal loss that has hindered single-cell analysis.

The advancements presented in this work significantly enhance the capability of ambient MSI, providing a robust and reproducible method for single-cell imaging. By bridging the gap between ambient analysis and high-resolution molecular imaging, our approach paves the way for more detailed investigations into cellular heterogeneity and metabolic dynamics. This, in turn, holds promise for transformative applications in biomedical research, including the development of more precise diagnostic tools and targeted therapeutic strategies.

Beyond enabling single-cell resolution, our modular DESI integration strategy provides a flexible, broadly compatible platform for future implementations. The use of modified ion source frames as the mounting interfaces allows our setup to be deployed across a wide range of Thermo Scientific mass spectrometers, including the legacy and current models. In principle, our design can be implemented to many other types of mass spectrometers using modified ion source interfaces and standard components such as motorized stage system and optical breadboard. A standard Thermo FAIMS system can be readily integrated to this design for improved detection sensitivity. Our design ensures that the method is not only effective but also scalable, adaptable, and immediately useful to a wide community of researchers in biomedical mass spectrometry.

## Supplementary Material



## Data Availability

Raw data of the DESI experiments can be obtained from the MassIVE database (MSV000095958).

## Notes

Experimental details of cell culture, cell attachment, and cell washing; MS spectrum; experimental parameters; experimental devices; photos of DESI-MSI experiment setups (PDF)
