# Supplementary material for: Achieving Single-Cell Resolution via Desorption Electrospray Ionization Mass Spectrometry Imaging (DESI-MSI) on Different Platforms
Source: Anal Chem. 2026 Mar 30;98(14):10629–38. doi: 10.1021/acs.analchem.5c08032 (PMC13084628; doi:10.1021/acs.analchem.5c08032)
Supplement: Supplementary file 1 [file ac5c08032_si_001.pdf]

Supporting Information

Achieving Single-cell Resolution via Desorption Electrospray Ionization Mass Spectrometry Imaging (DESI-MSI) on Different Platforms

Nathan Colwell,[1] Dan Chen,[1] Deepti Bhusal,[1] Zongkai Peng,[1] and Zhibo Yang[1,2]*

[1]University of Oklahoma, 101 Stephenson Parkway, Norman, OK 73019, USA

[2]Department of Biochemistry and Physiology, University of Oklahoma Health Campus, Oklahoma City, OK, 73104, USA

***CORRESPONDING AUTHOR:**
Zhibo-Yang@ou.edu

Table of Contents

Cell Culture and Preparation: ……………………………………………………………………S3

Cell culturing details……………………………………………………….……………..S3

Attaching cells to grids…………………………………………………..……………..S3

Cell washing procedure……..……………………..…………………………….………..S3

Cell Lysate preparation and coating on substrate……………..……..………..…………S3

Experimental Setup and Results: ……..…………………………….…………………….S4

Custom-built ion transfer capillary for Orbitrap XL ….…………………………….…..S4

Waters DESI XS/Thermo Exploris 240..………..…………………………..…………..S5

Custom-built ion transfer capillaries for Exploris 240 and Fusion Lumos…………..…..S6

Calibration curves for Limit of detection (LOD) measurement……………………………S7

Representative Spectra and MS/MS: ………………………..…………….………………..S7

Representative mass spectrum of OVCAR-8 cells from DESI XS/Synapt G2-SI……….S8

Representative mass spectrum of OVCAR-8 cells from DESI-XS/Orbitrap XL…………S8

Representative mass spectrum of OVCAr-8 cells from DESI-XS/Exploris 240…………S9

MS/MS spectra……………………………………………………………………..…….S9

Comparison of MSI results between Orbitrap XL and Exploris 240……………………..S11

Experimental Parameters: ……………………………………………….…….…………..S12

Mass Spectrometer Parameters…………………………………………………………S12

DESI XS Sprayer Parameters……………………………………………………….....S12

Ion Capillary Parameters………………………………………………………………….S12

Pixel Size Calculations……………………………………………………………………S12

Number of Features……………………………………………………………..……S12

Cell Culture and Preparation:

Cell Culturing Details. OVCAR-8 cells were cultured in tissue culture dishes (cat.no. FB012924, fisher, Pittsburgh, PA, USA) with RPMI-1640 medium supplemented with 10% fetal bovine serum (FBS) and 1% penicillin-streptomycin at 37°C in a humidified atmosphere with 5% CO_2 in the incubator (Heracell 150, Marshall Scientific, Hampton, NH, USA). Cells were passaged every 2-3 days upon reaching ~80% confluency to maintain exponential growth.

Attaching Cells to Grids. Upon reaching 80% confluency, OVCAR-8 cells were washed with phosphate buffer saline, detached using trypsin, and seeded onto a 6-well plate (product: 3471, Corning, NY, USA) containing gridded glass coverslips (cat.no:10817, ibidi, Gräfelfing, Germany). Approximately 5 x 10^5 cells were seeded per well, allowing cells to adhere and grow directly on the gridded glass coverslips. Cells were cultured for 12 hours to ensure complete attachment and appropriate confluency for subsequent single cell imaging experiments. Figure S2 shows ovarian cancer cells next to the grid, which allows for easy locating of the cells under the DESI setup. Each grid box is 50x50µm, which also allows us to quickly measure the size of the cells.

Cell Washing Procedure. Prior to imaging, the coverslips with cells were washed twice with 144 mM ammonium formate to remove any residual culture media and salts that could interfere with the mass spectrometry analysis. After washing, the cell-containing coverslips were dried in the air and placed on a glass slide (item# 1304, Globe Scientific, Mahwah, NJ, USA) and analyzed with DESI-MSI.

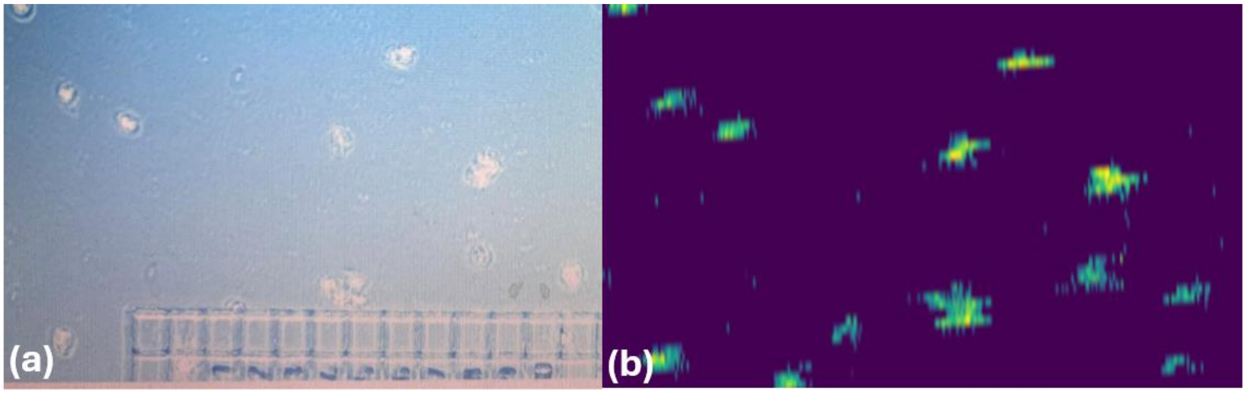

Figure S1. (a) OVCAR-8 cells attached grid glass cover slip. The grid (50 µm x 50 µm) and labels (i.e., letters and numbers) were used to locate single cells. (b) A representative MS image (m/z 239.106) of single cells with correlated locations. MSI experiments were conducted using the Waters DESI XS/Thermo Exploris 240 system.

Cell lysate preparation and coating on substrate. After OVCAR-8 cells reached ~90% confluency, the old media was discarded, 2mL of Trypsin was added, and the dish was incubated for 3 minutes at 37°C. Then, 8mL of complete RPMI-1640 media was

added for a total volume of 10 mL (2mL Trypsin + 8mL media). The dish was then centrifuged at 1500 rpm for 5 minutes. After discarding the supernatant, the cell pellet was resuspended in 1mL of cold Dulbecco's Phosphate-Buffered Saline (DPBS). After resuspension, 3mL of chloroform/methanol (2:1) was added and the mixture was vortexed for 10 minutes on ice. This mixture was centrifuged at 1500 rpm for 5 minutes and the cell debris was discarded. The bottom layer was transferred to clean 1.8mL tubes and centrifuged again at 1500 rpm for 5 minutes. This cell lysate layer was aliquoted into new tubes (~100 uL each), dried in a Speedvac (SPD111V, Thermo, Waltham, MA, USA), and stored at -80°C until use. To use the cell lysate, we resuspended the lysate in room temperature methanol (~100 uL) and centrifuged at 10000 rpm for 4 minutes at 4°C. The supernatant was transferred to a new tube for use. Then, on PTFE glass slides (ref: 041-MICRO-44, Scientific Device, Des Plaines, IL, USA), 10uL of cell lysate was coated on multiple spots and dried in air (**Figure S2**). After drying, the slide was taken for DESI-MSI analysis.

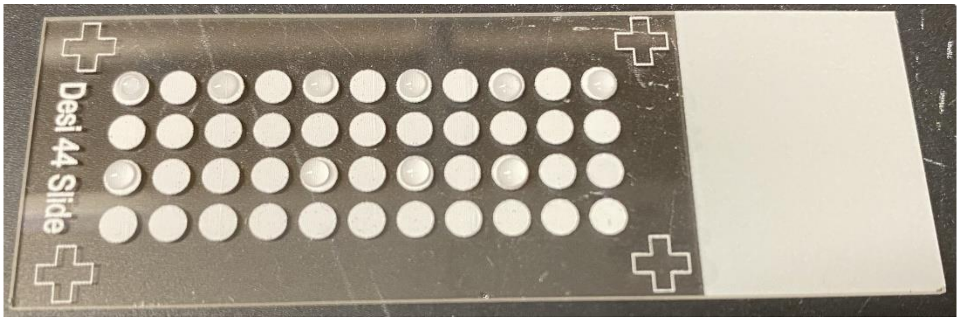

Figure S2. OVCAR-8 cell lysate droplets (10μL) on PTFE coated slide. The droplets were dried prior to analysis.

Experimental Setup and Results

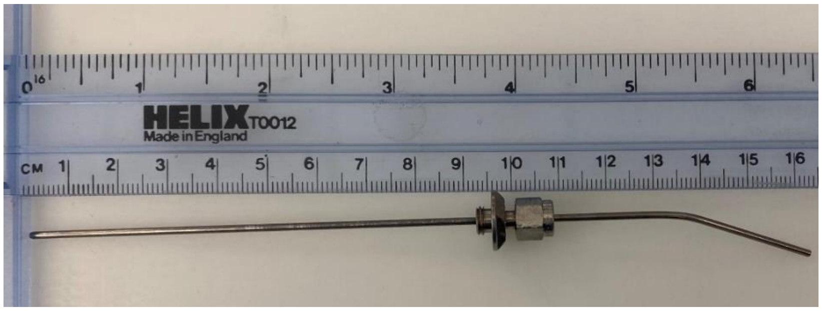

Figure S3. Custom-built ion transfer capillary used for (a) Waters DESI XS/Thermo Orbitrap XL and (b) Waters DESI XS/Thermo Orbitrap Fusion Lumos configurations.

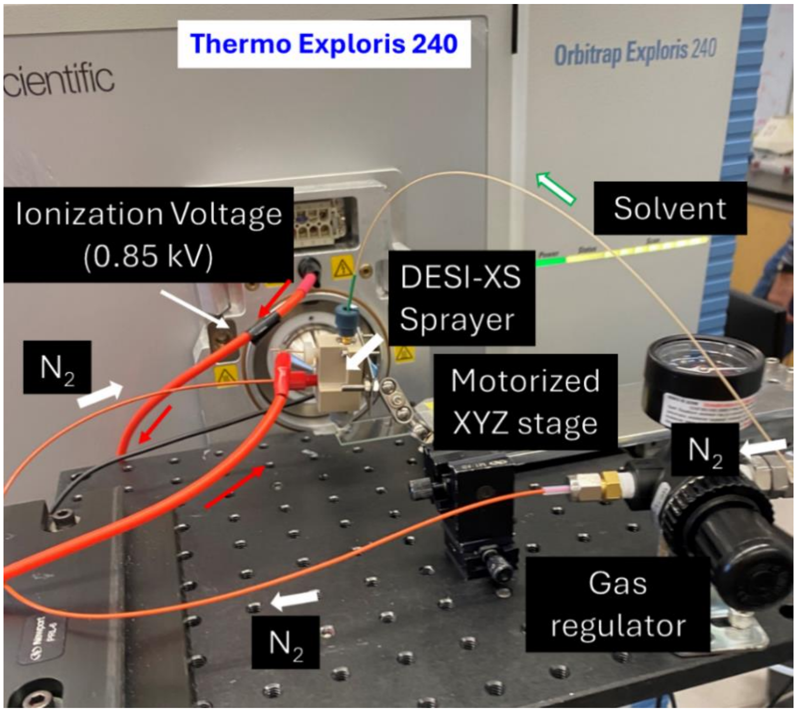

Figure S4. Coupling Waters DESI XS Sprayer to Thermo Exploris 240 Orbitrap via a home-built interface. N_2 is provided from bulding N_2 tubing and controlled by a gas regulartor. Solvent is provied by a Waters nanoAcquity UPLC pump.

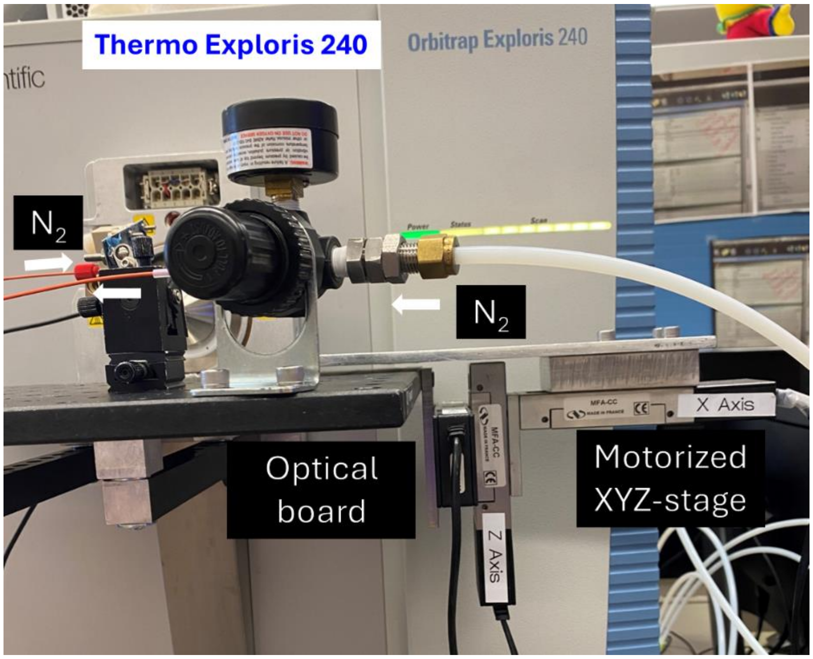

Figure S5. Motorized XYZ-stage for programmed cell sample movement. Cells were attached onto grid glass cover slips.

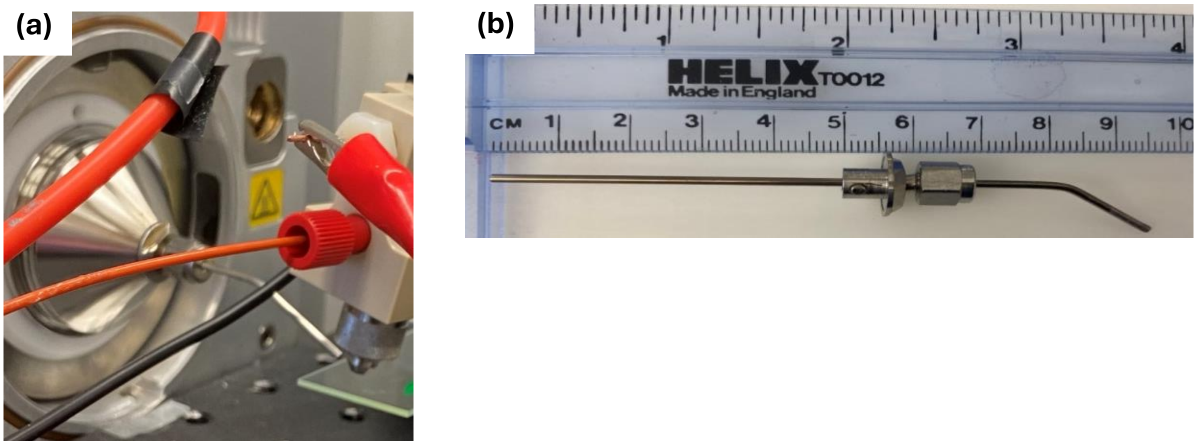

Figure S6. (a) Customized DESI XS/Exploris 240 interface and ion transfer capillary. The interface was converted from a Thermo FAIMS interface. (b) Customized ion transfer capillary used for the Waters DESI XS/Thermo Exploris 240 configuration.

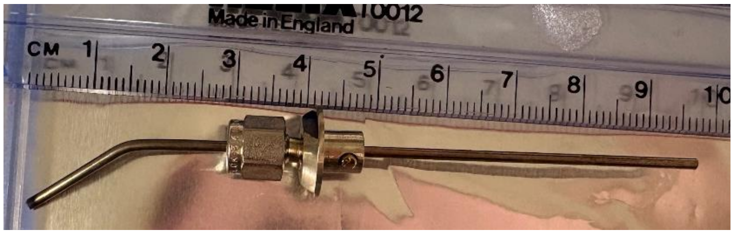

Figure S7. Customized ion transfer capillary used for the Waters DESI XS/Thermo Orbitrap Fusion Lumos configuration.

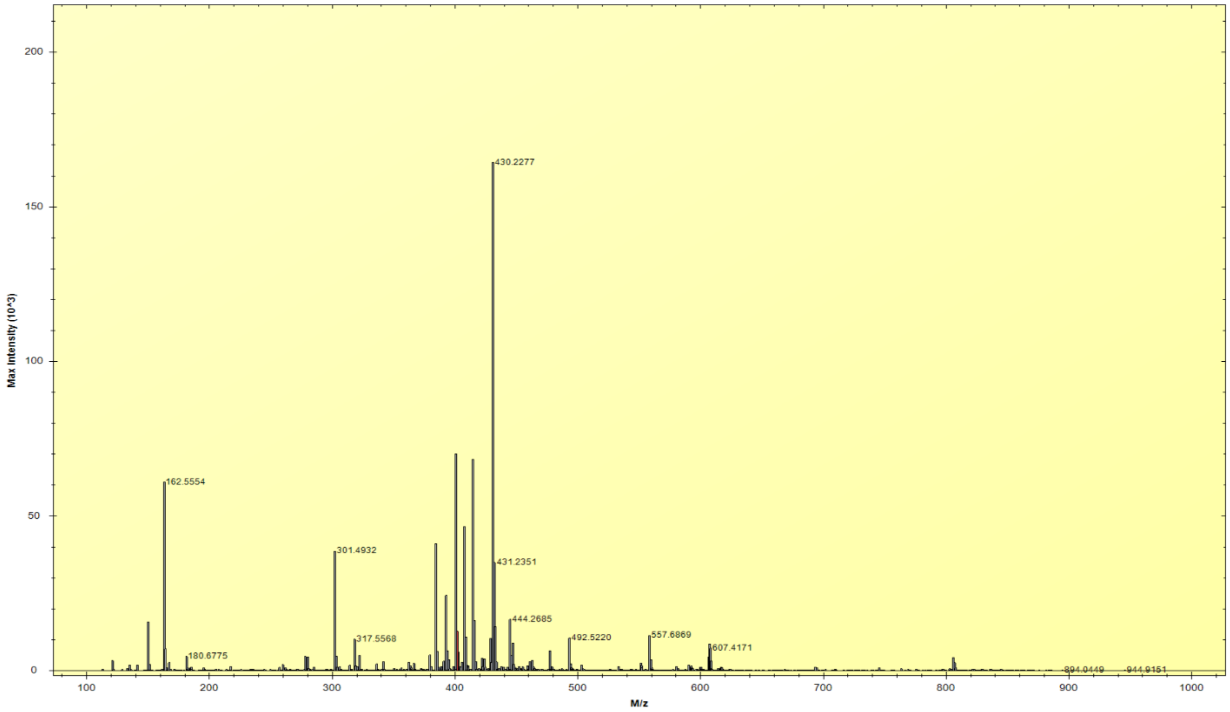

Figure S8. Representative mass spectrum of OVCAR-8 cells obtained from Waters DESI XS/Synapt G2-Si system.

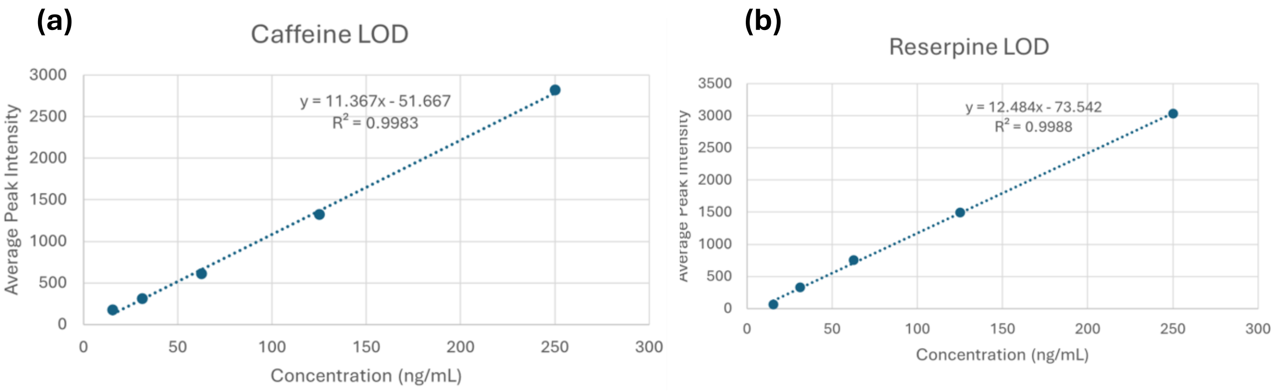

Figure S9. Calibration curve obtained from the DESI XS/Thermo Orbitrap Tribrid Fusion Lumos system for (a) caffeine and (b) reserpine. Plots were generated using the peak area (normalized level, NL) of the protonated caffeine (195.08) or reserpine (609.28) versus solution concentration. The calibration curve was used to determine the LOD of each compound.

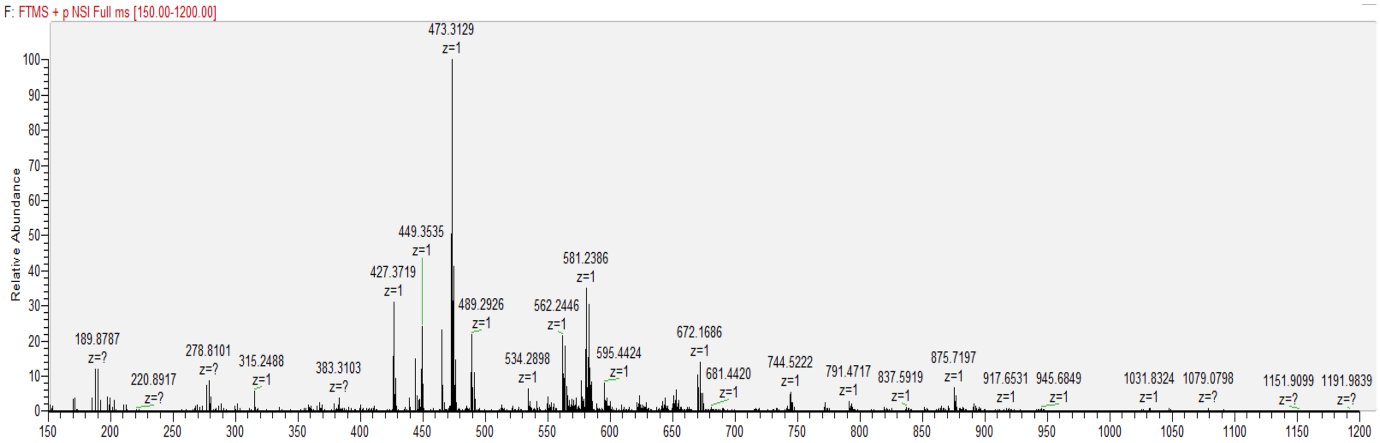

Figure S10. Representative mass spectrum of OVCAR-8 cells obtained from Thermo Orbitrap XL system.

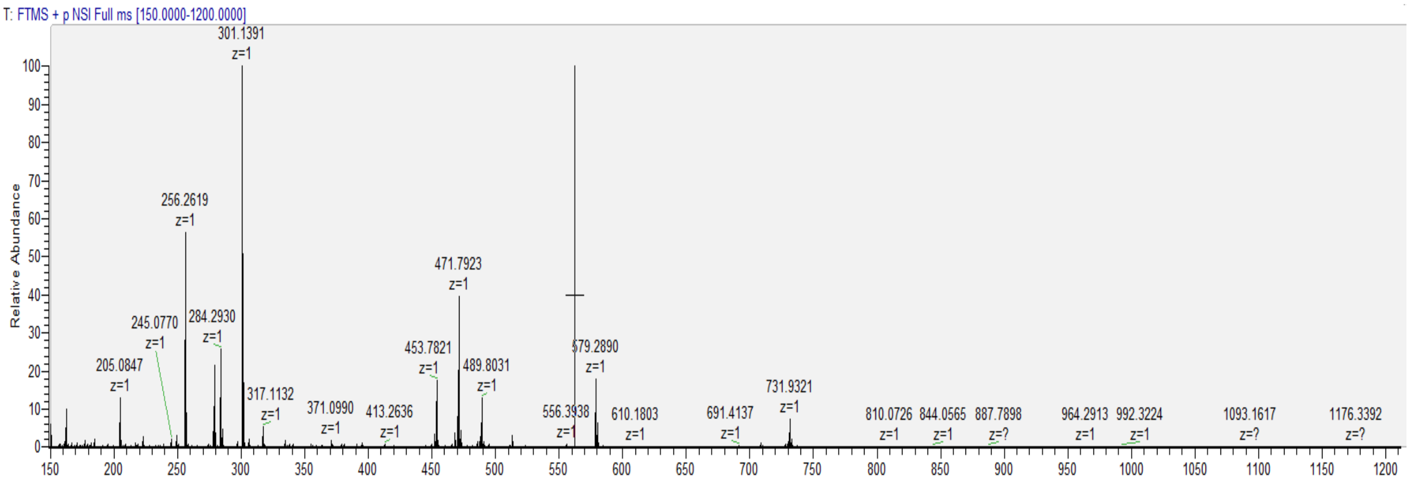

Figure S11. Representative mass spectrum of OVCAR-8 cells obtained from Thermo Exploris 240 system.

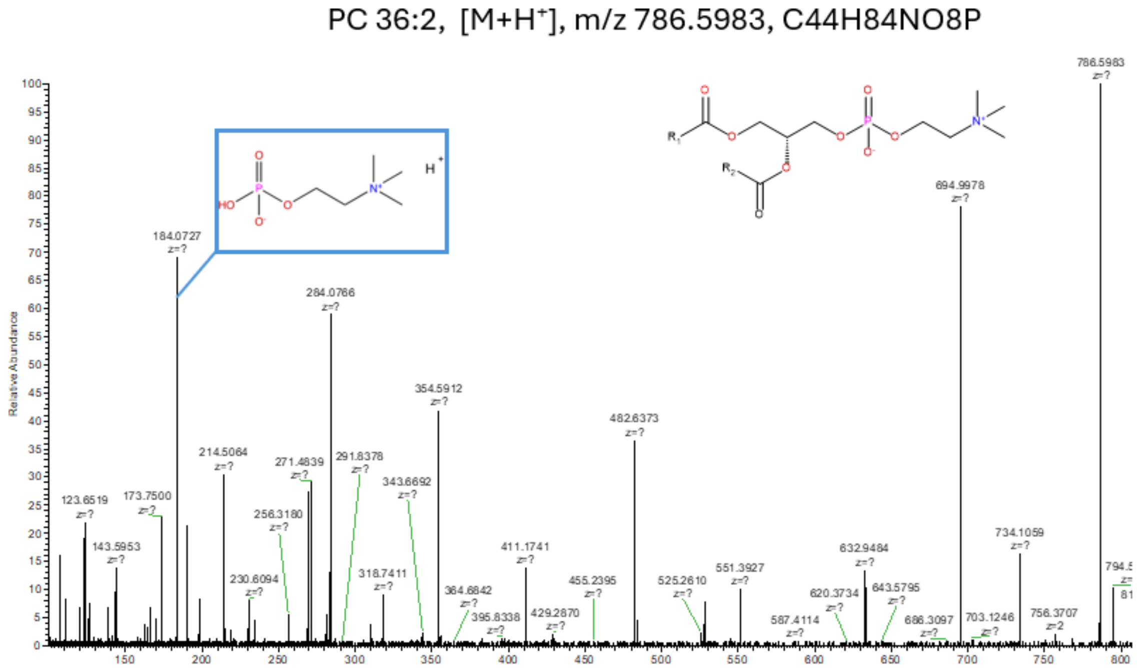

Figure S12. MS/MS spectrum of m/z 786.5983 (positive ion mode) obtained from Waters DESI XS/Thermo LTQ Orbitrap XL system.

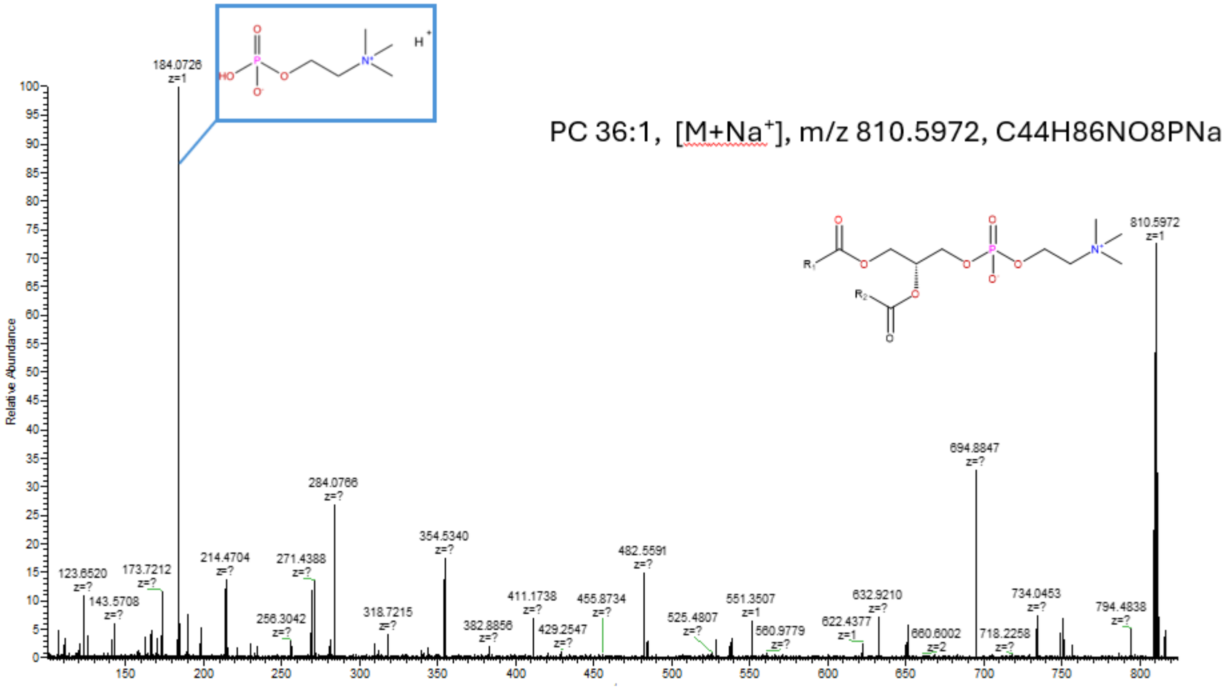

Figure S13. MS/MS spectrum of m/z 810.5972 (positive ion mode) obtained from Waters DESI XS/Thermo LTQ Orbitrap XL system.

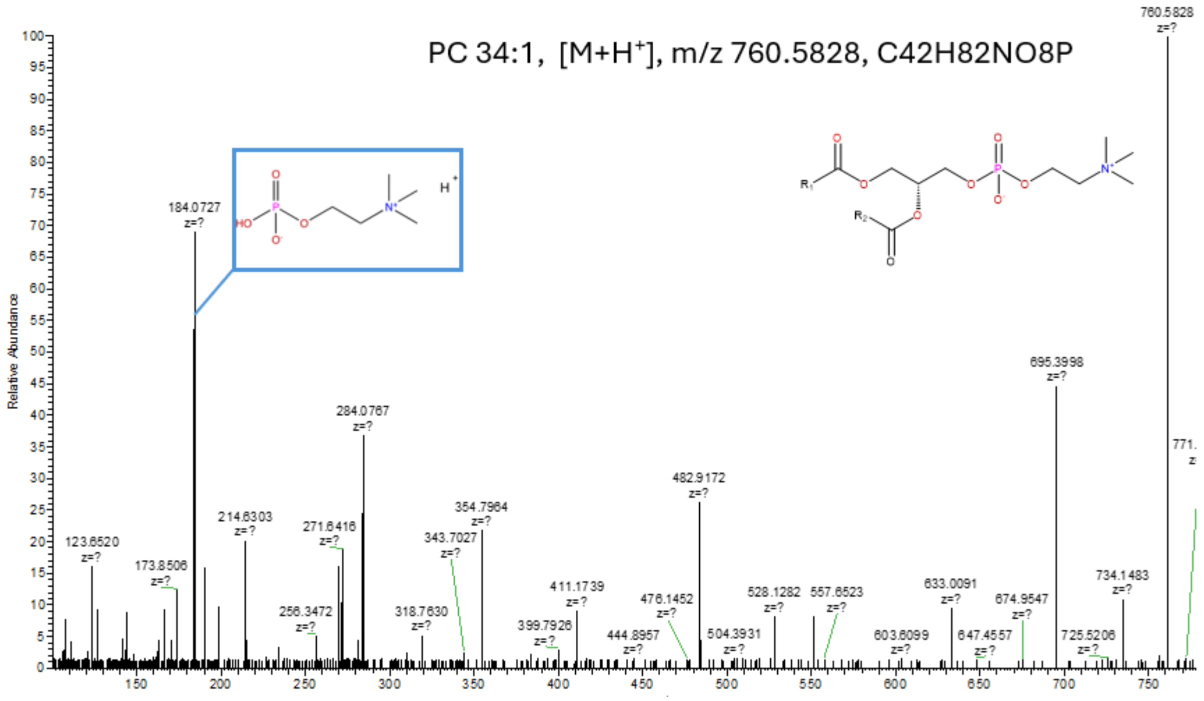

Figure S14. MS/MS spectrum of m/z 760.5828 (positive ion mode) obtained from Waters DESI XS/Thermo LTQ Orbitrap XL system.

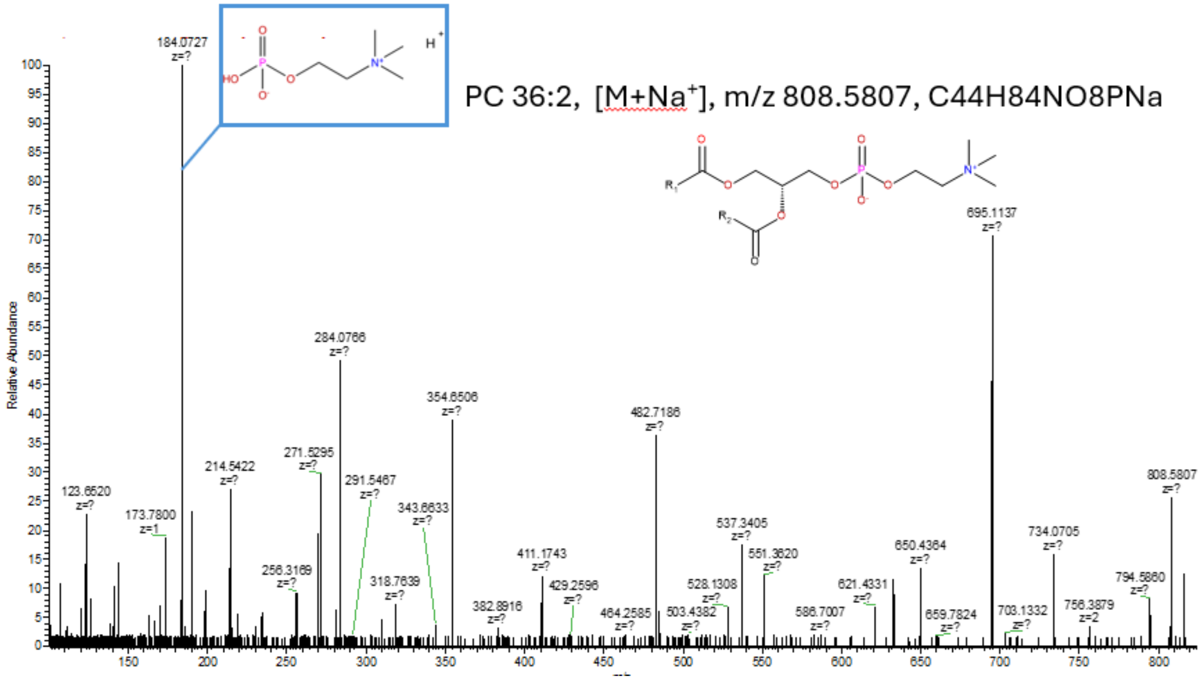

Figure S15. MS/MS spectrum of m/z 808.5807 (positive ion mode) obtained from Waters DESI XS/Thermo LTQ Orbitrap XL system.

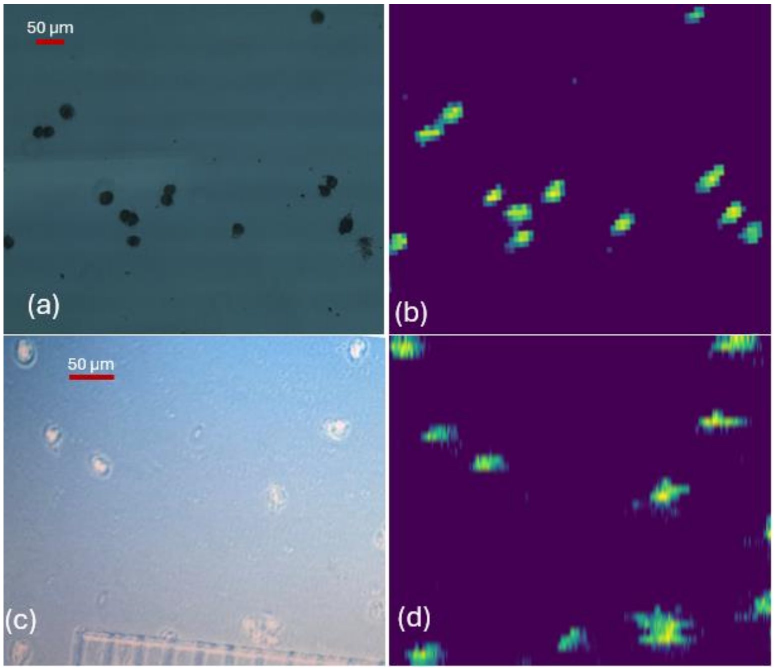

Figure S16. Comparison of MSI results between DESI/Orbitrap XL and DESI/Exploris 240 platforms. (a) Bright field microscopy photos and (b) the corresponding MS image (6.3 µm × 10 µm pixel size) of single cells measured using the DESI/Orbitrap XL platform. (c) Bright field microscopy photos and (d) the corresponding MS image (2.7 µm × 10 µm pixel size) of single cells measured using the DESI/Exploris 240 platform. Both MS images were generated using the same ion (m/z 786.59).

Experimental Parameters:

Mass Spectrometer Parameters. (1) The Waters Synapt G2-Si mass spectrometer was utilized in positive ion mode with an ionization voltage of +0.85kV. Settings of the mass spectrometer used include a mass range of m/z 100-1200, MS resolution of 20000, a pixel size of 10 µm x 10 µm, and a scan rate of 20µm/sec. (2) The LTQ Orbitrap XL was utilized in positive ion mode with an ionization voltage of +0.85kV with a mass range of m/z 100-1200, and a MS resolution of 30k. (3) The Exploris 240's parameters consisted of a positive ion mode with an ionization voltage of +0.85kV with a mass range of m/z 100-1200, and a MS resolution of 60k.

DESI XS Sprayer Parameters. The sprayer was positioned approximately 1.2 mm above the sample. The Synapt G2-Si setup consisted of a flowrate of 0.5 mL/min provided by the pump system of a Waters ACQUITY UPLC M-Class system (Waters Inc., Milford, MA, USA). For the LTQ Orbitrap XL and the Exploris 240, a solvent flowrate of 0.750 µL/min was utilized with a C18 column to increase backpressure (Waters, part no. 186009259, serial no. 04733426016309).

Ion transfer capillary Parameters. The stainless steel ion transfer capillary of the DESI XS device was positioned directly above the sample (~1 mm), and wrapped with heating wire for the Synapt G2-Si setup (Figure S1). For the heating wire, the voltage from our PSU (model: LGY-363000, Dong Guan Shi He Yu Tech, Dongguan, China) was set to 15.6V, which was the voltage required to reach and maintain 375°C, as read from the thermocouple. For the Orbitrap configurations, we used custom-built ion transfer capillaries to replicate the original ones (Figures S6-S7).

Pixel Size Calculations. Pixel size in MSI is defined by the distance the sample stage travels between consecutive mass spectra (stage velocity × acquisition time per scan) along X, and by the strip step along Y. For example, in the Exploris 240 system, 102 s ÷ 376 scans = 0.27 s/scan; at a stage velocity of 0.01 mm/s (10 µm/s), this corresponds to a 2.7 µm step in X. Combined with a fixed 10 µm strip step in Y, the resulting pixel dimension is 2.7 µm × 10 µm. Applying the same calculation yields pixel sizes of 6.3 µm × 10 µm for the LTQ Orbitrap XL configuration and 5.5 µm × 10 µm for the Thermo Orbitrap Tribrid Fusion Lumos.

Number of Features Obtained from Each Platform. Although MSI routinely detects a large number of ion features, a substantial portion originates from background ions associated with the substrate, solvent, or instrumental noise. Accordingly, the feature counts reported here represent approximate estimates of the true analyte-derived signals. To remove background-dominated features, a signal-to-noise (S/N) threshold of 3 was applied. For each detected feature, the median intensity across all cell-associated pixels was divided by the median intensity across all noise-only pixels. Application of this filter substantially reduced the number of retained features across all platforms, reflecting effective removal of background and instrumental signals. For the Synapt G2-Si dataset, ~70 features remained from the top 1,000 features selected during software-based peak picking. For the LTQ Orbitrap XL dataset, ~60 features remained from an initial set of 420 features. For the Exploris 240 dataset, ~720 features were retained from an initial 2,120 detected features.

Limit of Detection (LOD):

LOD values for caffeine and reserpine were determined from calibration curves generated using standards deposited (2.5 µL) onto PTFE-coated slides and analyzed by Waters DESI XS/Thermo Orbitrap Tribrid Fusion Lumos system. Blank solvent deposits were measured under identical conditions. The LOD was defined as the average blank signal plus three times the standard deviation of the blank ($I_{LOD} = \bar{I}_{blank} + 3\sigma_{blank}$). The concentration corresponding to this threshold was obtained by interpolation of the linear calibration curve (Figure S9).
